# Wireless Communications: Signal Processing Perspectives

**DOI:** 10.3390/e28010060

**Published:** 2026-01-02

**Authors:** Sébastien Roy

**Affiliations:** Department of Electrical and Computer Engineering, Université de Sherbrooke, Sherbrooke, QC J1K 2R1, Canada; sebastien.roy13@usherbrooke.ca

## 1. Introduction

Our global society is now tightly integrated with an interwoven technological web—the so-called digital fabric. People routinely interact with, and depend on, a vast distributed computational substrate composed of countless heterogeneous devices—smartphones, sensors, servers, embedded processors, cloud platforms, and edge nodes—all interconnected through an assortment of global networks. Among these interconnections, the wireless segment at the edge plays a uniquely critical role. It is the enabling layer for mobility, for ubiquitous access through handheld or wearable devices, and for the immense scale of machine-type communications that define the Internet of Things (IoT). In addition to familiar technologies such as Wi-Fi networks, low-power IoT radios like LoRa, and satellite links, there are now more than ten billion active cellular subscriptions—exceeding the number of people on the planet.

Yet, achieving high-rate communication over the air remains inherently challenging. The radio-frequency spectrum is a finite resource that becomes increasingly crowded as demand grows. The ongoing evolution in wireless communication has been driven in large part by increasingly clever and efficient spectrum usage rather than spectrum expansion. The recent relentless growth in capacity is in large part derived from advances in signal and spectrum processing, particularly those that harness the spatial domain. More specifically, since the mid-2000s, dramatic improvements in spectral efficiency have been realized through the use of adaptive antenna arrays and multiple-input/multiple-output (MIMO) techniques. Massive MIMO, in particular, has become one of the central architectural pillars of 5G networks.

Going forward, data traffic volume will continue to increase rapidly, as will the logistic complexity of wireless networks, which are becoming increasingly heterogeneous and unpredictable. Furthermore, there is a push for ultra-reliable and low-latency communications, which imposes further constraints on the wireless infrastructure. In fact, the need for extremely low-latency responses implies that much of the processing will be pushed towards the network edge, thus radically changing the nature of the wireless domain and its cybersecurity aspects.

Meeting these challenges requires continuous innovation in the signal processing domain to continue leveraging the spatial dimension with increasing efficiency in conjunction with other techniques to yield the desirable traits of ultra-reliability, ultra-low latency, self-organization, scalability, security, and adaptability to changing environments, operating conditions, and network demands. The scope of this Special Issue covers such innovations and the underlying challenges.

### 1.1. Spatial Processing and Decentralized Networks

The seminal paper by Marzetta [1] introduced the concept of *massive MIMO* (mMIMO) in 2010, generating immediate interest and numerous other papers (e.g., [2,3,4,5,6]). It is a departure from the previous multi-user MIMO (MU-MIMO) systems in that a large number of antennas at the base station serve a relatively small number of users/terminals per time/frequency resource block, i.e., the number of antennas is much larger than the number of addressed users. It demonstrates benefits with very large numbers of antennas such as the vanishing effects of noise, fading, and multi-user interference, while transmit power goes to zero and very simple linear processing becomes optimal. mMIMO has gone on to become a key enabling technology for 5G networks, essential to reach the performance targets.

However, practical mMIMO systems deployed for 5G networks have too few antennas to truly achieve the massive effect. In fact, they are considerably *more* power hungry than previous generations of base stations and require more sophisticated signal processing. Numerical processing complexity as well as RF circuit size and cost remain major hurdles. While useful, these systems are still far from capturing the full potential of the mMIMO concept. Furthermore, mMIMO techniques are inherently limited by the so-called *pilot contamination* problem and are sensitive to channel estimation imperfections.

While antennas themselves can be relatively simple and produced at low-cost, the associated RF chains can be problematic. In basic implementations of mMIMO, the need to have as many RF chains as antenna elements leads to excessive cost, size, and energy consumption. In practice, this ultimately limits the total number of achievable antenna elements. Indeed, RF chains comprise multiple analog and RF components (which do not follow Moore’s law), as well as A/D and D/A converters, which are significant bottlenecks.

Thus, various techniques have and continue to be explored to design large arrays where the number of RF chains is considerably smaller than the number of antennas. This includes antenna selection and hybrid beamforming [7], as well as exotic antenna technologies, such as holographic [8] and dynamic metasurface antennas [9]. With the latter, the degrees of freedom are achieved via fine control of the antenna in the analog domain rather than large numbers of RF chains.

Looking ahead, wireless systems must contend with escalating data traffic and rising operational complexity. Networks are becoming more unstructured and heterogeneous, incorporating a mixture of devices, mobility patterns, frequency bands, and deployment contexts. Moreover, new application classes—ranging from industrial automation to immersive communication—demand communication links with extremely high reliability and exceedingly low latency. Meeting such latency constraints often requires moving a significant portion of the computational burden from the cloud to network edge nodes, thereby profoundly transforming both the structure and the security landscape of future wireless systems.

Addressing these pressures calls for further advances in signal processing. Techniques that exploit spatial structure will need to evolve further and be combined with complementary approaches in order to deliver properties such as ultra-reliability, ultra-low latency, autonomous network behavior, scalability at massive device counts, and robustness to continually shifting channels, interference conditions, and traffic demands.

One of the key high-level trends to address these multiple challenges is a tendency towards decentralized, self-organized, user-centric, and amorphous architectures. Various forms of this are being explored for both basic network functionality (e.g., cell-free MIMO; see below) and security (e.g., blockchain [10], distributed authentication [11]).

*Cooperative MIMO* refers to techniques where multiple distributed wireless nodes coordinate their transmissions or receptions so that, collectively, they behave like a virtual multi-antenna array. By sharing data or channel information, these nodes can achieve spatial diversity, beamforming gains, and interference mitigation similar to conventional MIMO systems—without requiring all antennas to be co-located. This enables improved reliability, extended coverage, and higher throughput in networks where individual devices may only have a small number of antennas. It is also variously designated as *network MIMO*, *distributed MIMO*, *virtual MIMO*, and *virtual antenna arrays*.

Historically, cooperative MIMO evolved from simpler so-called macrodiversity schemes. These involve coordination and information sharing among a set of neighboring cells to better manage and limit inter-cell interference, particularly that associated with users near cell edges (which require a lot of power in both uplink and downlink). Primitive versions of this include dynamically choosing the best base station for a user at a cell edge depending on prevailing channel and interference conditions, and coordinating resource allocation for cell edge users to avoid interference. In 2000, the more radical idea of *joint transmission* was proposed whereby multiple base stations coordinate to transmit simultaneously to a cell edge user in such a way that their signals add up constructively at said user [12]. This is very challenging to achieve as the base stations must coordinate very quickly and precisely to ensure phase coherence at the targeted user. This was standardized in 2012 as the joint processing flavor of *coordinated multi-point (CoMP)* in LTE release 11.

Also, *cooperative diversity* was introduced in 2001. This is a multi-antenna technology which relies on combining two or more copies of the desired signal to achieve a diversity gain, where one copy is the direct signal, while the other(s) is(are) relayed copies in a multi-hop network arrangement. This implies time-deferred processing and the existence of relays, i.e., intermediate wireless nodes which receive the transmit signal, process it in some way, and forward it to the receiver. The said processing can be as simple as amplification (*amplify-and-forward*) or as involved as full decoding, modulation, and re-encoding (*decode-and-forward*). While this can greatly improve reliability, it sacrifices some degree of spectral efficiency.

Neither joint processing nor cooperative diversity leverage the power of spatial multiplexing. Thus, cooperative MIMO was introduced in 2001–2002 as a generalization of both, which fully exploits MIMO processing and its spatial multiplexing advantage [13].

In a related evolution of mMIMO which can be considered a generalization of cooperative MIMO, the concept of cell boundaries can be removed altogether. Indeed, the “massive” effect can be achieved in a more amorphous context (a departure from the rigid cell-based organization) by distributing the antennas across multiple access points (APs) which cooperate to serve a set of users. This is designated *cell-free massive MIMO* (CF-mMIMO) and such architectures significantly enhance user throughput and spectral efficiency, while minimizing issues such as shadow fading [14,15,16]. However, as is the case for cooperative MIMO, the coordination of multiple APs can be difficult to achieve due to excessive signaling and overhead, thus compromising the scalability of this approach [17], in particular, in the challenging and ultra-dense network environment foreseen for 6G [18]. As an example of related research, Andrea Goldsmith’s group at Princeton is pushing the envelope towards decentralized PHY and MAC layers in 6G networks, looking at, e.g., reducing the overhead of coordination [19] and the impact of imperfect coordination links [20].

### 1.2. Other Relevant Trends

The concept of physical-layer security dates back to the classic paper on the wiretap channel by Wyner [21]. It established that perfect secrecy is achievable if unauthorized users do not have knowledge of the authorized users’ channel, or the channel of unauthorized users is more noisy than the latter. Originally considered more academic than practical, the concept has become increasingly attractive for wireless channels [22]. Indeed, wireless channel coefficients in a rich multipath environment can be considered random, and being located just a few wavelengths away from the receiver means that a completely different and decorrelated channel is seen by an eavesdropper due to changing phase relationships among multiple paths. Thus, transmitter and receiver share a secret random number (the channel) known only to them, which can serve as a basis for a key.

Besides physical-layer security, it has been known since the birth of information theory that secrecy could be achieved by making the intercepted signal statistically indistinguishable from white background noise. One classical way to achieve this was through spread spectrum communications, whose predominant use until the 80s was for military secure communications, including robustness against jamming [23]. However, given their long history of usage, spread spectrum techniques are no longer sufficient to ensure secure exchanges when faced with the modern cyberthreat landscape. One alternative garnering significant attention is noise-based communication, first proposed in the early 2000s [24]. It was first demonstrated that it was possible to transmit a useful signal at zero signal power by simply modulating the thermal noise through resistors [25,26]. Such techniques, commonly and collectively designated *TherMod*, were later generalized to include active, artificial generation of information-carrying Gaussian noise signals (*NoiseMod*) [27].

Another active and related area is that of physical-layer authentication [28]. Such techniques can be roughly divided into active and passive schemes. Among passive approaches, uniquely identifying a device can be achieved through its RF *fingerprint*, which stems from various hardware imperfections which are detectable in the transmitted signal [29].

Artificial intelligence (AI) and machine learning (ML) are finding many uses in modern communication networks, and wireless communications is no exception [30]. Various forms of pattern recognition (such as the RF fingerprinting mentioned above) are obviously natural applications for AI. Other common problems where AI shines involve complex multi-objective optimization, including radio resource allocation and routing in multi-hop networks, where there is no clear global optimum. These are typically non-convex, NP-complete problems where traditional analytical approaches fare poorly.

Historically, the appetite for more spectrum has pushed systems to increasingly higher bands, as evolutions in RF hardware progressively made it possible. Thus, communications in the Terahertz band (0.1–10 THz) are becoming increasingly attractive [31] due to very large available bandwidth, potentially reduced power consumption, and diminutive antenna sizes, among other things. The THz band, located squarely in the spectrum between millimeter waves and infrared, remained largely unused until devices appeared capable of generating and detecting them in the 1980s [32]. In recent years, a number of laboratory prototypes have demonstrated the feasibility of coherent THz communications [33]. However, as pointed out in one of the contributions in this Special Issue, there are few analytical performance results in the literature thus far, given the very recent emergence of these systems.

## 2. Contributions

This Special Issue is dedicated to research that pushes the frontiers outlined above, highlighting both the innovations required and the fundamental challenges that accompany them. It comprises 10 contributions, of which 9 are regular research papers and one is an extensive 74-page review paper.

Among these, six relate to some form of spatial processing, with three of them focusing specifically on massive MIMO, while the review paper tackles cooperative MIMO. Of those six, the remaining two papers are concerned with beamforming techniques, with one of them leveraging beamforming for physical-layer security. In addition to the latter, two other papers deal with secure or covert wireless communications, with one of them focusing on RF fingerprinting (authentication at the physical layer). The latter explores a ML approach, which is natural given that fingerprinting is essentially a pattern recognition problem.

In the remaining two papers rounding out the issue, one is concerned with ML techniques as a basis to construct a clustering and multi-hop routing protocol in wireless sensor networks (WSNs). Finally, the last paper provides an analysis of error performance of BPSK modulated coherent Terahertz wireless links in line-of-sight channels with pointing errors.

The following discusses the contributions organized by theme, while the numbering reflect the sequence of presentation in the issue.

### 2.1. Papers Related to Spatial Processing

The paper by Hou et al. (contribution 2) proposes an evolution of the classic direction-of-arrival (DOA) estimation method, MUSIC (MUltiple SIgnal Classification), which traditionally uses an antenna array to estimate the directions of arrival of multiple impinging signals. MUSIC, which dates back to 1986, has been well studied and, while exhibiting high DOA precision (so-called *super-resolution*) under the right circumstances, it breaks down in the presence of multipaths due to its sensitivity to coherent sources, i.e., multiple wavefronts carrying essentially the same signal. While other DOA methods that do not suffer from this drawback exist, MUSIC remains attractive due to its simplicity and effectiveness. Thus, various augmentations or modified versions have been proposed over the years to overcome this salient drawback. The most common approach consists in performing a form of pre-processing called *spatial smoothing* prior to applying MUSIC.

In this context, contribution 2 explores the application of MUSIC in beamspace, while relying on a single RF chain and a dynamic metasurface antenna (DMA), instead of the classical linear antenna array. This allows dynamic optimization of the beams in order to focus more specifically on a Region Of Interest (ROI) and thus reduce computational effort while maintaining precision. To reduce numerical complexity, the 2D estimation problem is broken down into two 1D problems (azimuth and elevation). The authors combine multiple techniques in order to achieve 2D DOA estimation in a multipath-robust fashion relying on a single RF chain, including (a) using a time-division data reception scheme and associated pilot structure to gather the necessary estimation data with a single RF chain, while leveraging the rapid switching capability of the DMA, (b) application of blind source separation (BSS) in order to decipher the multipath signals, and (c) application of 1D-MUSIC in both dimensions. This is an innovative combination of techniques which, thanks to the flexibility of the DMA, exhibits effectiveness at a reasonable computational cost and has the capability of dynamically focusing on a given ROI, while requiring only a single RF chain.

Contribution 3 by Almeida et al., presents a novel analytical treatment of performance assessment in cell-free user-centric mMIMO networks. Therein, it is shown for the first time how the Beckmann distribution can be applied to represent the effective channel gain experienced by the *k*th User Equipment (UE) on the downlink of such a network. By further approximating the multi-user interference (MUI) as Gaussian noise, simple expressions are derived for the outage probability, normalized ergodic capacity, and maximum transmission rate. As a prior step underpining the latter expressions, the Probability Density Function (PDF) and Cumulative Density Function (CDF) of the Signal-to-Interference-plus-Noise ratio (SINR) are also derived. It is further shown that, irrespective of the distribution of the channel gains between APs and UEs, the effective gain follows a Beckmann distribution. Simulation results show close agreement with their analytical counterparts. This contribution constitutes a significant step forward in the fundamental understanding of key performance metrics in this important class of networks.

Contribution 4 by Yang et al. proposes a specific Deep-Learning (DL) method for improving channel estimation in mMIMO systems. In fact, channel estimation can often be considered a weakness of mMIMO systems since their performance in both uplink and downlink is highly dependent upon the accuracy of Channel-State Information (CSI). However, given the large number of antennas involved, the computational burden of channel estimation can quickly become prohibitive. Thus, researchers strive to develop methods that strike an appropriate balance between computational complexity and accuracy; in recent years, this has included various ML approaches, which complement traditional methods based on estimation theory. Restricting our attention to pilot-based methods (as opposed to blind and semi-blind estimation algorithms), traditional schemes include many variants of the Least-Squares (LS) and Minimum Mean-Square-Error (MMSE) estimators. While the LS approach is less computationally demanding, it is less precise and more sensitive to noise than the MMSE one. More recently, multiple estimation methods based on DL were proposed, and most consider the 2D channel matrix as an image, thus casting the problem into the well-studied problem of image denoising. This is the case for the method proposed here as well, which combines features of traditional methods and DL as a means to achieve high accuracy at low complexity. Extensive simulations demonstrate that the Normalized Mean-Square Error (NMSE) accuracy is superior to both traditional and DL-based reference methods, while the ccomplexity is lower than that of the MMSE estimator.

The second paper by Fu et al. (contribution 8) deals with both beamforming and security, since location-based beamforming is used here as a physical-layer security component. It considers that the transmitter (Alice) is equipped with an array having a relatively large number *M* of antennas (simulation results are given for values of *M* between 8 and 100), the legitimate receiver (Bob) has a single antenna, and the eavesdropper (Eve) is equipped with a smaller array of *N* antennas, with *N* in results ranging from 1 to 8. Line-Of-Sight (LOS) propagation is assumed between both Alice and Bob and Alice and Eve; hence, multipath fading envelopes are presumed to follow the Rice distribution. The basic premise is that, provided the AP has a sufficiently large number of antennas, and given knowledge of Bob’s location, it can steer a precise beam in his direction. Thus, the hope is that eavesdroppers would hear this signal as very faint and drowned in noise, and the communication would therefore be relatively secure. However, there is the possibility that the eavesdropper (Eve) is sufficiently close to Bob to be illuminated in part by the beam. Thus, security can be improved by also sending an aritifical noise (AN) signal into a beam targeted at Eve, thus reducing her effective SINR. In effect, this reduces the area around Bob where eavesdropping is feasible. The paper, assuming that the AP has knowledge of the locations of both Bob and Eve, provides a solid analytical treatment of the performance based on some approximations. Closed-form approximate PDFs of the SINRs are derived, as well as the expectations of the SINRs and BERs. Numerical results show good agreements with the analysis. They also show that the proposed beamforming approach significantly degrades the performance of nearby eavesdroppers, even with a small number of antennas at the AP.

The paper by Wang (contribution 9) explores a method for nearly optimal detection of high-order constellations in mMIMO links. According to the rich literature on the mMIMO detection problem, the maximum-likelihood method is largely considered to be optimal, but its exponential complexity is considered prohibitive. At the other end of the spectrum, linear detectors using the MMSE or the Zero-Forcing (ZF) criterion achieve low complexity, but at the cost of a significant performance penalty (in terms of output SINR or error rate). Various other methods have been proposed which fill the gap between these two extremes, such as MMSE-SIC (Successive Interference Cancellation) and various local search approaches, with the latter attempt being an approach to the performance of maximum-likelihood while limiting complexity by restricting the search space in the lattice to a region “likely” to contain the optimal point according to some heuristics. These include sphere decoding and likelihood ascending search. However, the authors mention that high-order constellations exacerbate the detection problem, and will greatly expand the size of the search space in these local search methods. Methods based on variational inference, where the true likelihood function is approximated by a distribution which is easier to manipulate, constitute yet another class of detection solutions. Two such methods, namely the Gaussian Tree Approximation (GTA) and Expection Consistency (EC) methods, are combined here by the author in a nested alternance to (a) formulate a general framework based on so-called *nested variational chains* where they show that many existing algorithms, including MMSE, GTA, and EC, can be considered special cases within said framework; and (b) formulate a new GTA-EC algorithm based on this framework which is shown to achieve improved detection performance at lower complexity than existing algorithms.

Contribution 10 by Favier and Rocha constitutes a major review paper (74 pages), taking the form of a tutorial on tensor-based methods applied to cooperative MIMO. As its title suggests (“Part 2”), it is the sequel of a previous paper (Part 1) by the same authors published in 2023, also in *Entropy*. Part 1 deals with tensor-based models for cooperative MIMO in general, while Part 2 focuses on semi-blind receivers for MIMO one-way two-hop relay systems, where such receivers can jointly estimate both the transmitted symbols and the channels while requiring relatively few pilot symbols. The general nature of the cooperative MIMO concept, as well as its importance in future wireless networks, is well established and was discussed above in subsection I-A. By allowing very general, multi-dimensional modeling of various systems, tensors have seen increased usage in signal processing over the last two decades. As a fundamental analytical tool, they provide multiple advantages, including natural and unified representation of multi-dimensional systems and phenomena, the possibility of capturing sparsity in multiple dimensions as a reduced rank subspace, as well as very flexible and general factorizations which naturally allow joint estimation of multiple parameters. The latter are especially useful for joint estimation in blind or semi-blind communication scenarios, and it is this core capability of tensor factorizations which is studied at length in this paper. The latter, i.e., the “Part 2” paper, which constitutes contribution 10, is itself broadly divided into two parts, where the first part (consisting of sections 2–4) addresses tensor operation, models, and algorithms, with a specific focus on the nested decompositions NTD (Nested Tucker Decomposition) and NCPD (Nested Canonical Polyadic Decomposition). This part aims to provide a self-contained introduction to tensor models applicable to the design of wireless communication systems. It notably includes a unified and original presentation of NTD and NCPD, based on a graph representation. Meanwhile, the second part (sections 5–7) presents an overview of two-hop relay systems and semi-blind receivers. Section 5 first provides an overview of cooperative and two-hop relay systems, while Section 6 is concerned with semi-blind receivers. Starting from a general overview of the latter, it also proposes two classes of semi-blind algorithmes. Finally, Section 7 provides simulation results comparing the various algorithms discussed.

### 2.2. Papers Related to Secure Wireless Networks

The paper by Zhou et al. (contribution 1) explores a particular modulation method that makes the transmitted signal look like noise without structure, thus making it exceedingly difficult to decode without prior knowledge, essentially based on phase randomization. As noted therein, classic spread spectrum techniques are no longer sufficient to ensure covert communication given the increased sophistication of attacks. What is proposed here is a form of OFDM (Orthogonal Frequency Division Multiplexing) where, in each block, each subcarrier is assigned an independent random phase. This causes the resulting signal to look very unstructured and noise-like. Furthermore, the scheme includes a cognitive radio component as it first senses the propagation environment and only assigns subcarriers to subbands that are deemed “unoccupied,” i.e., whose power is below a given threshold. Cyclic Code Shift Keying (CCSK), well-known from spread-spectrum secure communications, is also integrated into the method to increase its robustness against eavesdropping. From a very practical standpoint, the authors note that random phase systems such as this one tend to produce very high peak-to-average power ratios (PAPRs) which is detrimental in several ways, including power efficiency. They therefore apply various post-processing techniques (signal mapping, selective mapping, and signal clipping) to limit the PAPR. Simulation results indicate that the resulting signal spectrum closely approximates that of white noise. The time domain waveforms also behave like random noise, ensuring that a receiver without prior knowledge of the transmission parameters cannot effectively detect the signal.

The first paper by Fu et al. (contribution 7) consists of an in-depth study of Radio Frequency Fngerprint (RFF) identification for various models of 5G mobile phones. RFF identification is a topic of current interest which sees a significant amount of research activity, while being greatly relevant for various security problems. Broadly speaking, it is the art of identifying a brand and specific model of cellphone or wireless transmitter strictly based on certain observable characteristics of its transmitted waveform, which stem from specific hardware imperfections. Thus, it has been demonstrated in the literature that it is possible in practice to identify with high accuracy the transmitting device based solely on this unique “signature” or fingerprint. The introduction of the paper provides an overview of the various hardware imperfections which can be leveraged for this purpose, while justifying their choice of carrier frequency offset behavior (CFO) as the observable basis. In the proposed method, the raw In-line/Quadrature (I/Q) data from the RF chain is first preprocessed into a stable 2D representation, the differential constellation trace figure (DCTF). The latter was introduced in a previous paper in 2019 (where the first author, L. Peng, is the last author here) and it consists of a 2D representation of the differential evolution of a signal time series which can be leveraged to extract fingerprint information without a priori synchronization. Here, the DCTF results are fed to a series of convolutional neural networks (CNNs) where each is trained on a particular 5G preamble format, since there are 64 such PRACH (Physical Random Access CHannel) formats in 5G New Radio (NR). Results show that the proposed method can reach an identification accuracy of 92.78% with six devices, and its performance is shown to be superior to other methods reported elsewhere. Interestingly, the said results are based on an actual experimental setup with an Open Air Interface (OAI) base station running on a software radio platform, and six actual cell phones of three different models.

### 2.3. Other Papers

To achieve self-organization benefits, large-scale wireless networks need to deploy some form of distributed intelligence applicable to resource allocation. An example of this is contribution 5 by Wang et al. which proposes such a distributed approach to tackle dynamic clustering and multi-hop routing in WSNs, with the goal of maximizing the lifespan of the network when the nodes powered by batteries. This is a typical WSN scenario, where a relatively large number of nodes is distributed randomly to perform a given sensor task in environments where battery replacement may be difficult or impossible. With the passage of time, the batteries necessarily become depleted, and the nodes stop functioning at different times due to unequal task-related or mainly routing-related loads. In this context, the network is considered alive while there is still a sufficient density of nodes which are functioning. Maximizing this lifespan is closely related to the routing strategy, which can be dynamically fine-tuned to better balance battery depletion across the node population. Clustering is an approach which can reduce energy consumption, whereby the node population is divided into local clusters with one node in each being elected Cluster Head (CH). In combination with multi-hop routing, nodes route their packets first to their CH, which then forwards them to the base station directly, or through one or more other CH, depending on the distance to cover. Dynamically electing CHs based on their residual energy allows a certain energy balancing, but does not address the fact that nodes near the base station will naturally be more solicited. In unequal clustering, this is mitigated by making the cluster size increasingly smaller as one approaches the base station. Thus, this paper proposes a method which combines fuzzy logic and Q-learning to jointly optimize unequal clustering and multi-hop routing. Simulations show that the scheme achieves significantly improved network lifetime compared with two existing algorithms. It also exhibits significant improvement in terms of network stability and overall throughput.

Contribution 6 by Niu et al. is concerned with the error performance of coherent THz communications. While THz communications have become of practical interest since the 1980s, the initial focus was on non-coherent demodulation, given the difficulty of detecting phase at such high frequencies. However, recent laboratory prototypes have demonstrated the feasibility of coherent demodulation. As this is a recent development, insightful analytical results are still quite rare. In this paper, the authors provide exact and asymptotic analytical results for BER and outage probability of coherent, Binary Phase Shift Keying (BPSK) LOS THz links, taking into account the two main performance impediments in such, namely atmospheric turbulence and pointing errors. As THz wave behavior is quasi-optical, transmitter and receiver are typically equipped with a highly directional antenna to form the equivalent of a Free-Space Optical (FSO) link. The pointing error then refers to the misalignment between receiver detector and the incoming beam, and this can be modeled statistically. A previous effort by the first two authors and others provided similar analytical results with turbulence and pointing error, but was restricted to non-coherent THz links. In addition to exact results, contribution 6 also provides asymptotic analytical expressions, which, due to their simplicity, are very insightful. Numerical results show that in the high SNR region, exact and asymptotic BER results are in close agreement.

## 3. Conclusions

The papers in this Special Issue paint a cohesive story, which speaks to the relevance of current signal-processing-related developments in modern and future wireless networks. The representation of forms of spatial processing is dominant, reflecting their unquestioned importance in wireless communication evolution. This is intertwined with an observed and necessary drift towards more decentralized and ad hoc network organizations, such as in cell-free or user-centric systems, as well as wireless sensor networks. Secure communications based on physical-layer security is a parallel trend, which is likely to receive increasing interest given the challenges raised by decentralization. This is especially true when the communication devices are limited in computing resources and/or power, such as in machine-to-machine, Internet-of-Things, or wireless sensor networks communications. Also, the push for more spectrum and for greener energy efficiency will motivate the further development of THz radio links.

Finally, it can be observed that various forms of artificial intelligence and machine learning show benefits in many wireless communication functions, even in areas where traditional algorithms were solid and considered optimal in some sense. It is interesting to note that in many cases, superior performance can be obtained by combining machine learning and traditional approaches, such as those demonstrated in contributions 4 and 5.

This story will be continued in a sequel Special Issue currently in preparation.
